# Calf Compartment Syndrome associated with the Use of an Intra-osseous Line in an Adult Patient: A Case Report

**DOI:** 10.5704/MOJ.1611.014

**Published:** 2016-11

**Authors:** R Malhotra, WL Chua, G O’Neill

**Affiliations:** Department of Orthopaedics, National University Hospital Singapore, Singapore

**Keywords:** Compartment syndrome, intra-osseous line, intraosseous infusion

## Abstract

We present a case of a lower limb compartment syndrome associated with the use of an intra-osseous line inserted into the proximal tibia in an adult patient. An unconscious 59-year old male with multiple injuries presented to our Emergency Department after a road traffic accident. Bilateral proximal tibial intra osseous-lines were inserted due to poor venous access. After resuscitation his left leg was noted to be tense and swollen with absent pulses. Acute compartment syndrome was diagnosed both clinically and with compartment pressure measurement. Two incision fasciotomy on his left lower leg was performed. Intra osseous-lines in the proximal tibia are increasingly used in adult patients in the pre-hospital setting by paramedics and emergency physicians. Their use, along with the possible complications of these devices, such as the development of compartment syndrome or osteomyelitis leading to amputation, is well reported in the paediatric literature. To the best of our knowledge, there have not been any previous reports of complications in the adult patient. We present a case of lower leg compartment syndrome developing from the use of an intra-osseous line in the proximal tibia in an adult patient. With the increasing use of intra-osseous lines in adult patients, clinicians should be aware of the possibility of developing compartment syndrome which may lead to disability or amputation in severe cases.

## Introduction

We present a case of an obtunded patient involved in a road traffic accident who developed compartment syndrome from extravasation of fluids infused via a left tibial intra-osseous line. As far as we are aware this is the first report of compartment syndrome in an adult from use of an intraosseous line.

## Case Report

A 59-year old male pedestrian was hit by a vehicle on an expressway. He was brought to the Emergency Department and immediately intubated due to a low Glasgow Coma Scale Score. He underwent resuscitation according to ATLS protocol and underwent fluid and blood product resuscitation. Venous access was poor, therefore, intraosseous lines were inserted into both proximal tibiae for infusions. Into his left tibia intra-osseous line, two units of packed red cells (approximately 500 mls in volume) and 1000 mls of crystalloid were infused over a period of two hours. The right tibia line was not used.

After initial computer tomography scans from head to pelvis the following injuries were identified: Subarachnoid haemorrhage with frontal lobe haemorrhagic contusions; multiple facial bone fractures; left pulmonary contusion, pneumothorax with left flail chest; left iliac wing pelvic open fracture with sacro-iliac joint disruption and an overlying degloving injury and left clavicle fracture.

After urgent angioembolisation of the distal branches of his left superior gluteal artery and distal branches of his iliolumbar arteries, he underwent insertion of an intracranial pressure monitor and venous cut down for vascular access to substitute the intra-osseous lines.

At the end the surgery the patient’s left leg was notably tense and swollen. The orthopaedic team reviewed him in the operation theatre and clinical examination revealed a tense and cool leg, non palpable distal pulses, with no external clinical evidence of fractures or soft tissue contusion. Compartment pressure was measured at 199mmHg for the deep posterior, anterior and lateral compartments and 140mmHg for the superficial posterior compartment.

**Fig. 1:**
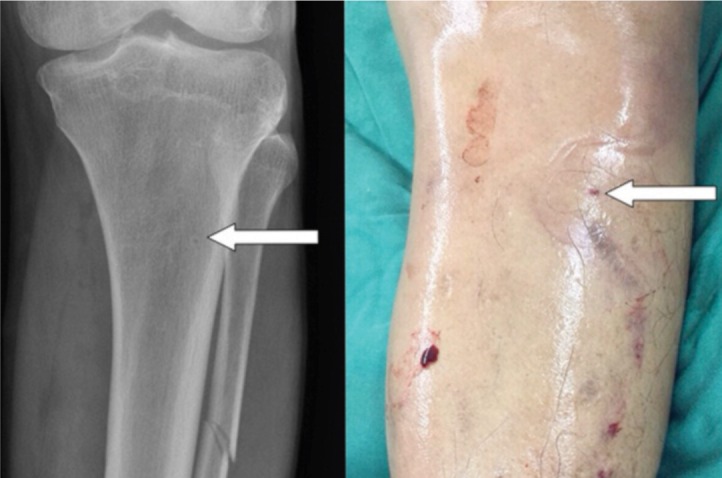
Radiograph and clinical photograph of swollen left leg prior to fasciotomy.

A standard two-incision fasciotomy with four compartment decompression was performed immediately revealing approximately 500ml of blood clots and bulging calf muscles which were contractile and viable. There was no obvious source of active bleeding that needed hemostatic control. Distal pulses returned following the fasciotomy. A plain radiograph of his left tibia showed a non-displaced fibula shaft fracture which was treated non-operatively. The clinical picture prior to initial fasciotomy and the subsequent radiograph are shown. (Fig. 1)

After two further debridements, his leg fasciotomy wounds were closed without skin grafting. During his two-month inpatient stay, he achieved full neurological recovery. However, due to the pelvic injury, he still required walking aids six months after the accident. There was no long term complication directly linked to his compartment syndrome or its treatment.

## Discussion

Intra-osseous lines in the proximal tibia are increasingly used in adult patients in the pre-hospital setting by paramedics and emergency physicians. The medullary canal of bone contains a vascular plexus that communicates directly with the vascular system of the limb involved, providing a direct path from the medullary canal to the central circulation; but dislodgement or poor insertion technique may lead to extravasation. Complications from intra-osseous lines although infrequent includes compartment syndrome and osteomyelitis. These complications can result in serious consequences such as amputation^1^. Compartment syndrome following use of intra-osseous lines has been reported in the paediatric literature,^2,3^ but there are no reported case in the adult population.

Compartment syndrome is diagnosed both clinically and with compartment pressure measurement. Alert patients will complain of pain out of proportion to the clinical situation, with signs of swelling and pain on passive stretch of muscles. If prolonged, signs include paraesthesia, paralysis and pulselessness. Compartment pressures with an absolute value of 30mm Hg and higher, or higher than a relative value of 30mm Hg below the diastolic pressure can be diagnostic of compartment syndrome^4^. In acute compartment syndrome of the calf, treatment is two-incision fasciotomy of the leg, incising the fascia enclosing all four compartments of the lower leg. However, if the diagnosis is delayed, or the presentation is late, muscle necrosis and nerve damage may have already occurred which can lead to primary amputation of affected limb.

Fortunately in our patient the tense limb was noted early and appropriately managed. This highlights the importance of repeating secondary surveys in trauma patients, especially in the unconscious patient, to assess for evolving pathology or previously missed injuries.

The underlying cause of his compartment syndrome could include soft tissue injury, fracture or re-perfusion injury. The latter is not relevant here but he did have a fibula shaft fracture. Fibular fractures from ballistic injuries resulting in compartment syndrome have been reported in the literature ^5^. There were no signs of soft tissue contusion or laceration and the fracture was not comminuted or displaced which would indicate a high energy impact typically leading to compartment syndrome. Nonetheless, his leg was already at risk of compartment syndrome due to the fibular fracture but the compartment pressure of 199mmHg was much higher than usually encountered in acute compartment syndrome. This would almost certainly be due to the extravasation of large amounts of infusion into the local compartments, considering the large volume of haematoma.

With the increasing frequency of using intra-osseous lines in the adult patient, it is crucial for the attending physician to recognize the possible complication of compartment syndrome developing from their use.
